# Pump–Probe Optical Response and Four-Wave Mixing in a Zinc–Phthalocyanine–Metal Nanoparticle Hybrid System

**DOI:** 10.3390/mi14091735

**Published:** 2023-09-04

**Authors:** Natalia Domenikou, Spyridon G. Kosionis, Ioannis Thanopulos, Vassilios Yannopapas, Emmanuel Paspalakis

**Affiliations:** 1 Materials Science Department, School of Natural Sciences, University of Patras, 26504 Patras, Greece; domenikou.n@gmail.com (N.D.); kosionis@upatras.gr (S.G.K.); ithano@upatras.gr (I.T.); 2 Department of Physics, School of Natural Sciences, National Technical University of Athens, 15780 Athens, Greece; vyannop@mail.ntua.gr

**Keywords:** pump–probe optical response, nonlinear optical response, four-wave mixing, two-level quantum system, zinc–phthalocyanine molecule, metallic nanoparticle

## Abstract

We investigate theoretically the optical response of a zinc–phthalocyanine molecular quantum system near a gold spherical nanoparticle with a radius of 80 nm. The quantum system is irradiated by a strong pump and a weak probe coherent electromagnetic field. Using the density matrix methodology, we obtain analytical expressions for the absorption, dispersion, and the four-wave-mixing coefficients. The influence of the nanoparticle on the spontaneous decay rate of the quantum system, as well as on the external fields, are obtained by an electromagnetic Green’s tensor method. The spectroscopic parameters of the molecule are also obtained by ab initio methods. For the studied optical spectra, we find that, below a critical distance between the molecule and the plasmonic nanoparticle, determined by the minimal value of the effective Rabi frequency, single-peaked spectra are observed. Above this critical distance, the spectra exhibit the characteristic Mollow-shaped profiles. The enhancement of the pump field detuning induces the shift of the sideband resonances away from the origin. Lastly, and most importantly, regardless of the value of the detuning, the optical response of the system is maximized for an intermediate value of the interparticle distance.

## 1. Introduction

In recent studies, several methods for manipulating the nonlinear optical response of quantum systems have been applied in order to enhance the light–matter interaction at the nanoscale. In particular, it has been demonstrated that the enhancement of the optical response of a quantum system can be realized by coupling it with a metallic plasmonic nanostructure. It has been further shown that, due to the efficient interaction between the exciton and the localized surface plasmons, nonlinear optical phenomena such as the induction of nonlinear Fano absorption resonances [1,2,3,4,5,6,7,8,9], nonlinear optical rectification [10,11,12,13], optical transparency [14], gain without inversion [15,16,17,18], Kerr nonlinearity [19,20,21,22], and optical bistability [23,24,25] can be substantially modified.

These investigations have been conducted mainly on nonlinear optical processes in two-level quantum systems near plasmonic nanostructures. Several works have examined the pump–probe response and the nonlinear four-wave mixing (FWM) effect of a two-level quantum system near a plasmonic nanostructure, when the system interacts with a weak probe field and a strong pump field. Specifically, Li and Zhu studied the creation of modified cross-Kerr nonlinearity [19] and slow light [26]. Sadeghi studied lasing without inversion and explored the effects of plasmonic metaresonances on the absorption/gain spectrum of the probe field [15,17]. Moreover, Li et al. analyzed the cross-Kerr nonlinearity and showed optical bistability effects [27] as well as studied the effect of four-wave parametric amplification [28]. Paspalakis et al. studied the modified FWM spectrum [20] while Kosionis and Paspalakis studied the absorption and dispersion properties of the probe field under the influence of the pump field [29] as well as the control of the self-Kerr nonlinearity [21] and stressed the connection between plasmonic metaresonances and bistability and their effect on the studied spectra. The FWM has been also studied by Singh et al. [30].

In the previously mentioned studies [15,17,19,20,21,26,27,28,29,30], a small spherical metallic nanoparticle (MNP) was considered, typically with a radius up to 10 nm, and the theoretical treatment considered the effects of exciton–plasmon coupling and electric field modification in the quasistatic regime. The effects of modification of the decay rate of the quantum system (Purcell effect), which was a quantum dot in most cases, on the nonlinear optical response were not studied. In the present study, we analyze the pump–probe response and the FWM effect on a zinc–phthalocyanine molecule [31], modeled as a two-level quantum system, near a relatively large gold nanosphere. The zinc–phthalocyanine molecule is chosen as it has potential for large nonlinear optical coefficients [31] and also has a resonance near the plasmon peak of the studied MNP. The two-level system is described using realistic molecular spectroscopic parameters obtained by ab initio methods. The MNP we study has a radius of 80 nm, so the quasistatic approximation cannot be applied. We use an electromagnetic Green’s tensor technique for the computation of the electric field modification factor and the quantum system’s Purcell factor, which affects the two-level system’s spontaneous decay rate, due to the presence of the MNP in the environment of the molecule. More specifically, we assume that the hybrid system coherently interacts with a strong pump and a weak probe electromagnetic field, both being tangentially polarized with respect to the surface of the MNP. We obtain the probe field susceptibility in the first order and the FWM coefficient under the influence of the strong pump field, which is studied in all orders. We next vary the distance between the quantum system and the surface of the MNP for different values of the pump field detuning from resonance in order to study its impact on the dispersion/absorption and FWM of the quantum system.

The paper is structured as follows: In Section 2, starting with the density matrix equations, we derive analytical expressions for the FWM coefficient as well as for the real and the imaginary part of the optical susceptibility of the probe field under the presence of the pump field when the two-level quantum system is located near the MNP. In Section 3, we first present the spectroscopic parameters of the molecular quantum system obtained by ab initio methods as well as the field modification and Purcell factors obtained using the Green’s tensor method. Next, we investigate the impact of the distance between the surface of the MNP and the quantum system on the dispersion, absorption, and FWM spectra of the quantum system. The conclusions of our study are summarized in Section 4.

## 2. Theory

**Figure 1:**
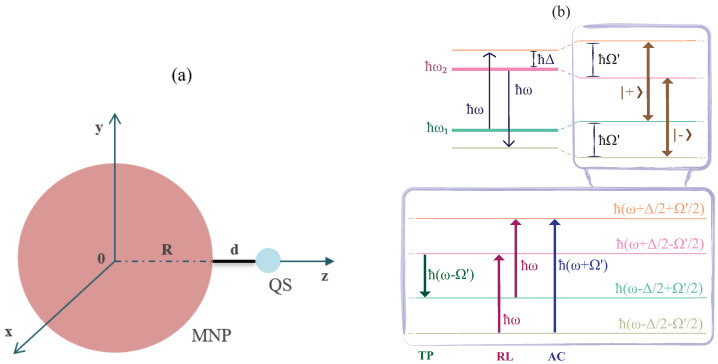
(**a**) Schematic depiction of a quantum system located at a distance *d* from the surface of the MNP. (**b**) Energy level scheme of the dressed states, |+〉 and |−〉, and the transitions between them in the case of negative detuning $\Delta =\omega -\omega_{21}$. TP, RL, and AC stand for the three-photon resonance process, the stimulated Rayleigh scattering process, and the AC-Stark effect, respectively. $\Omega^{\prime }=\sqrt{\Delta^{2}+{\left|\Omega_{a}\right|}^{2}}$ denotes the effective Rabi frequency.

In this work, the system under investigation, which is shown in Figure 1a, is modeled as a two-level quantum system and a spherical MNP, similar to previous studies [15,17,19,20,21,26,27,28,29,30], but in this case with larger MNP radius, specifically R=80 nm. The distance *d* is defined as the distance between the quantum system and the surface of the MNP. The energy levels of the quantum system are represented as |1〉 and |2〉, while the transition energy is $\hbar \omega_{21}$ and the induced element of the dipole moment associated with the transition is denoted by $\mu_{21}$. Specifically, here we consider the energetically lowest rovibrational state of the ground and first singlet electronic states of the molecule as the two energy levels of the two-level system. This is a good approximation here, as the two electronic states of the molecule are energetically well separated (about 2 eV), and in low (cryogenic) temperatures, where coherent nonlinear optical experiments are performed, only the rovibrational ground level of the ground electronic state is populated. Further, in this work, we assume that the quantum system has a transition dipole moment along the *x*-axis, tangentially to the surface of the MNP.

**Figure 2:**
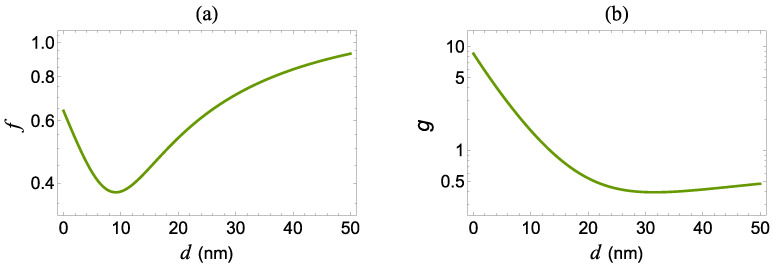
(**a**) The field modification factor of the field, *f*, and (**b**) the Purcell factor, *g*, as a function of the distance *d* between the quantum system and the surface of the Au-MNP for energy equal to 1.9445 eV, which corresponds to the transition frequency $\omega_{21}$ of the molecular quantum system.

The system described above interacts with a coherent electric field of the form,

(1) $$\begin{array}{cc} \vec{E}\left(t\right) & =\left[E_{a}e^{-i\omega t}+E_{b}e^{-i(\omega +\delta )t}+c.c\right]\hat{x} \end{array}$$

with polarization direction $\hat{x}$. We assume that the applied electric field excites the transition between the two levels of the quantum system and it also excites surface plasmons on the MNP, which, in turn, affect the optical response of the quantum system and the actual form of the electric field felt by the quantum system. In Equation (1), the electric field is composed of a strong pump field with frequency ω and amplitude $E_{a}=fE_{a}^{f}$ and a weak probe field with frequency ω+δ and amplitude $E_{b}=fE_{b}^{f}$. $E_{a}^{f}$ and $E_{b}^{f}$ are the field amplitudes in the absence of the MNP and $\delta =\omega_{b}-\omega$ denotes the frequency mismatch between the two applied fields. The factor *f* is the field modification factor due to the interaction of the quantum system with the MNP located in its vicinity. The field modification factor *f* expresses the modification of the incident field due to scattering from the MNP and essentially provides the electromagnetic field at the position of the quantum system [32,33]. The field modification factor is presented in Figure 2a as a function of the distance *d* between the quantum system and the surface of the Au-MNP for energy equal to 1.9445 eV, which gives the transition energy $\hbar \omega_{21}$ of the molecular quantum system considered in this work. We also note that the field modification factor features a minimum at about d=9 nm. We note that, for the spherical Au-MNP, the modification factor *f* depends on the radius of the MNP and the resonance frequency considered.

The Hamiltonian that describes the dynamics of the quantum system coupled to the spherical MNP, while interacting with the applied electromagnetic fields, within the rotating wave and the dipole approximations, is given by

(2) $$\begin{array}{cc} \hat{H}\left(t\right) & =\hbar \omega_{1}\left|1\rangle \langle 1\right|+\hbar \omega_{2}\left|2\rangle \langle 2\right|\\ \; & -\frac{\hbar }{2}\left[\Omega_{a}e^{-i\omega t}+\Omega_{b}e^{-i(\omega +\delta )t}\right]\left|2\rangle \langle 1\right|+H.c., \end{array}$$

where $\Omega_{a}=f\Omega_{a}^{f}$, with $\Omega_{a}^{f}=2\mu_{21}E_{a}^{f}/\hbar$ being the modified Rabi frequency associated with the strong pump field. In analogy, we define the modified Rabi frequency $\Omega_{b}=f\Omega_{b}^{f}$ of the weak probe field, with $\Omega_{b}^{f}=2\mu_{21}E_{b}^{f}/\hbar$. Starting with the Liouville–von Neumann equation, we obtain for the slowly varying density matrix elements

(3) $${\dot{\rho }}_{21}\left(t\right)=\left(i\Delta -\frac{1}{T_{2}}\right)\rho_{21}\left(t\right)-\frac{i}{2}\left[\Omega_{a}+\Omega_{b}e^{-i\delta t}\right]\left[2\rho_{22}\left(t\right)-1\right],$$

(4) $${\dot{\rho }}_{22}\left(t\right)=-\frac{\rho_{22}\left(t\right)}{T_{1}}+\frac{i}{2}\left[\Omega_{a}+\Omega_{b}e^{-i\delta t}\right]\rho_{12}\left(t\right)-\frac{i}{2}\left[\Omega_{a}^{*}+\Omega_{b}^{*}e^{i\delta t}\right]\rho_{21}\left(t\right)\;.$$

In addition, we denote $\Delta =\omega -\omega_{21}$ as the detuning of the pump field from resonance, $T_{1}=1/\Gamma$ as the population relaxation time, and $T_{2}=2T_{1}/\left(1+2T_{1}\gamma_{d}\right)$ as the relaxation time due to dephasing processes with rate $\gamma_{d}$. The presence of the MNP alters the optical properties of the quantum system. First of all, it modifies the characteristic times $T_{1}$ and $T_{2}$ due to the Purcell effect; the spontaneous decay rate is now given by $\Gamma =g\Gamma^{f}$, with $\Gamma^{f}=\omega_{21}^{3}\mu_{12}^{2}/3\pi c^{3}\hbar \epsilon_{0}$ being the spontaneous decay rate in free-space. The factor *g* is the Purcell factor for a quantum system with *x*-oriented transition dipole moment; it is shown in Figure 2b as a function of the distance *d* between the quantum system and the surface of the MNP for energy equal to 1.9445 eV, which corresponds to the transition energy $\hbar \omega_{21}$ of the molecular quantum system considered in this work.

Both factors presented in Figure 2 are calculated via an electromagnetic Green’s tensor method. The dielectric constant of the Au-MNP of R=80 nm is taken from the experimental results of Reference [34] and the molecular quantum system is embedded in vacuum, i.e., $\epsilon_{e}=1$.

In order to investigate the first-order nonlinear optical effects with respect to the probe field, we assume that the amplitude of the pump field is much larger than the amplitude of the probe field, $|\Omega_{b}\left|\;\ll \;\right|\Omega_{a}|$. Thus, while all the orders of the interaction of the pump field with the quantum system are maintained in the calculations, we consider only first-order interaction terms between the probe field and the quantum system and truncate the higher-order ones. Thus, the steady-state solutions to the density-matrix elements are given by the following Taylor-series expansion equations

(5) $$\rho_{21}\left(t\right)=\rho_{21}^{\left(0\right)}\left(t\right)+\rho_{21}^{\left(-\right)}\left(t\right)e^{-i\delta t}+\rho_{21}^{\left(+\right)}\left(t\right)e^{i\delta t},$$

(6) $$\rho_{22}\left(t\right)=\rho_{22}^{\left(0\right)}\left(t\right)+\rho_{22}^{\left(-\right)}\left(t\right)e^{-i\delta t}+\rho_{22}^{\left(+\right)}\left(t\right)e^{i\delta t},$$

with $\rho_{21}^{\left(0\right)}\left(t\right)$ and $\rho_{22}^{\left(0\right)}\left(t\right)$ denoting the density matrix elements, in the limiting case when the quantum system exclusively interacts with the strong pump field [35]. The element $\rho_{21}^{\left(-\right)}\left(t\right)$ is related to the pump–probe optical response of the quantum system, while the element $\rho_{21}^{\left(+\right)}\left(t\right)$ describes the FWM process. We note that the terms $\rho_{22}^{\left(-\right)}\left(t\right)$ and $\rho_{22}^{\left(+\right)}\left(t\right)$ are related to the population pulsation of the quantum system. Since the population of the excited state is a real quantity, we deduce that the relations $\rho_{22}^{\left(0\right)}\left(t\right)=\rho_{22}^{(0)*}\left(t\right)$ and $\rho_{22}^{\left(-\right)}\left(t\right)=\rho_{22}^{(+)*}\left(t\right)$ must hold. The inequalities $|\rho_{21}^{\left(-\right)}\left(t\right)|,|\rho_{21}^{\left(+\right)}\left(t\right)|\;\ll \;|\rho_{21}^{\left(0\right)}\left(t\right)|$ and $|\rho_{22}^{\left(-\right)}\left(t\right)|,|\rho_{22}^{\left(+\right)}\left(t\right)|\;\ll \;|\rho_{22}^{\left(0\right)}\left(t\right)|$ must be satisfied, too.

By introducing the Equations (5) and (6) into Equations (3) and (4), we obtain the following set of differential equations,

(7) $${\dot{\rho }}_{21}^{\left(0\right)}\left(t\right)=\left(i\Delta -\frac{1}{T_{2}}\right)\rho_{21}^{\left(0\right)}\left(t\right)-i\Omega_{a}\left[\rho_{22}^{\left(0\right)}\left(t\right)-\frac{1}{2}\right],$$

(8) $${\dot{\rho }}_{21}^{\left(-\right)}\left(t\right)=\left(i\Delta +i\delta -\frac{1}{T_{2}}\right)\rho_{21}^{\left(-\right)}\left(t\right)-i\left[\Omega_{a}\rho_{22}^{\left(-\right)}\left(t\right)+\Omega_{b}\rho_{22}^{\left(0\right)}\left(t\right)-\frac{\Omega_{b}}{2}\right],$$

(9) $$\begin{array}{c} {\dot{\rho }}_{21}^{\left(+\right)}\left(t\right)=\left(i\Delta -i\delta -\frac{1}{T_{2}}\right)\rho_{21}^{\left(+\right)}\left(t\right)-i\Omega_{a}\rho_{22}^{\left(+\right)}\left(t\right), \end{array}$$

(10) $${\dot{\rho }}_{22}^{\left(0\right)}\left(t\right)=-\frac{\rho_{22}^{\left(0\right)}\left(t\right)}{T_{1}}+\frac{i}{2}\left[\Omega_{a}\rho_{21}^{(0)*}\left(t\right)-\Omega_{a}^{*}\rho_{21}^{\left(0\right)}\left(t\right)\right],$$

(11) $${\dot{\rho }}_{22}^{\left(-\right)}\left(t\right)=\left(i\delta -\frac{1}{T_{1}}\right)\rho_{22}^{\left(-\right)}\left(t\right)+\frac{i}{2}\left[\Omega_{a}\rho_{21}^{(+)*}\left(t\right)+\Omega_{b}\rho_{21}^{(0)*}\left(t\right)-\Omega_{a}^{*}\rho_{21}^{\left(-\right)}\left(t\right)\right].$$

When the system has reached its state of dynamic equilibrium, the time derivatives in Equations (7) and (11) are equal to zero and thus the steady-state analytical solutions of the elements $\rho_{21}^{\left(0\right)}$, $\rho_{21}^{\left(-\right)}$, $\rho_{21}^{\left(+\right)}$, $\rho_{22}^{\left(0\right)}$, $\rho_{22}^{\left(-\right)}$, and $\rho_{22}^{\left(+\right)}$ are obtained.

Based on these results, the effective linear susceptibility $\chi^{\left(1\right)}\left(\delta \right)$ is given by

(12) $$\begin{array}{cc} \chi^{\left(1\right)}\left(\delta \right) & =\frac{N\mu_{12}}{\epsilon_{0}E_{b}}\rho_{21}^{\left(-\right)}\\ & =\frac{2N|\mu_{12}{|}^{2}}{\epsilon_{0}\hbar }\frac{\left(\rho_{22}^{\left(0\right)}-\frac{1}{2}\right)\left[\left(\delta +\frac{i}{T_{1}}\right)\left(\delta -\Delta +\frac{i}{T_{2}}\right)-\frac{|\Omega_{a}{|}^{2}\delta }{2\left(\Delta -\frac{i}{T_{2}}\right)}\right]}{\left(\delta +\Delta +\frac{i}{T_{2}}\right)\left(\delta -\Delta +\frac{i}{T_{2}}\right)\left(\delta +\frac{i}{T_{1}}\right)-{\left|\Omega_{a}\right|}^{2}\left(\delta +\frac{i}{T_{2}}\right)}, \end{array}$$

with *N* being the number density of atoms.

The magnitude of the FWM effect is quantified by ${\left|\rho_{21}^{\left(+\right)}\right|}^{2}$; it is given by the following analytical expression:

(13) $$\begin{array}{cc} FWM\equiv {\left|\rho_{21}^{\left(+\right)}\right|}^{2}= & \frac{{\left(\Delta \delta -\delta^{2}+\frac{2}{T_{2}^{2}}\right)}^{2}+{\left(\frac{3\delta -2\Delta }{T_{2}}\right)}^{2}}{4\left[{\left(\Delta^{2}-\delta \Delta -\frac{1}{T_{2}^{2}}\right)}^{2}+{\left(\frac{2\Delta -\delta }{T_{2}}\right)}^{2}\right]}\times \\ \; & \frac{|\Omega_{a}{|}^{4}{\left|\Omega_{b}\right|}^{2}{\left[\rho_{22}^{\left(0\right)}-\frac{1}{2}\right]}^{2}}{{\left(\delta^{3}-\delta {\left|\Omega^{\prime }\right|}^{2}-\frac{\delta }{T_{2}^{2}}-\frac{2\delta }{T_{1}T_{2}}\right)}^{2}+{\left(\frac{\delta^{2}-\Delta^{2}}{T_{1}}+\frac{2\delta^{2}-{\left|\Omega_{a}\right|}^{2}}{T_{2}}-\frac{1}{T_{1}T_{2}^{2}}\right)}^{2}}, \end{array}$$

which depends on the amplitude and the frequency of both the pump field and the probe field [36]. In Equation (13), we have introduced the effective Rabi frequency $|\Omega^{\prime }|=\sqrt{\Delta^{2}+{\left|\Omega_{a}\right|}^{2}}$. Furthermore, the steady-state solution of the density-matrix element $\rho_{22}^{\left(0\right)}$ is given by

(14) $$\rho_{22}^{\left(0\right)}=\frac{|\Omega_{a}{|}^{2}T_{1}T_{2}}{2(1+\Delta^{2}T_{2}^{2}+|\Omega_{a}{|}^{2}T_{1}T_{2})}.$$

Evidently, as should be expected, the value of the density-matrix element $\rho_{22}^{\left(0\right)}$ depends only on the pump and not on the probe field.

In order to describe the dynamic optical response of the quantum system, we perform a dressed state analysis. For this purpose, we examine the response of the quantum system under the presence of a strong pump field only.

The Schrödinger equation is now written as $i\hbar |\dot{\Psi }\left(t\right)\rangle =\hat{H}|\Psi \left(t\right)\rangle$, where $\hat{H}$ represents the Hamiltonian of Equation (2) after removing the terms associated with the weak probe field, and $|\Psi \left(t\right)\rangle =c_{1}\left(t\right)e^{-i\omega_{1}t}+c_{2}\left(t\right)e^{-i\omega_{2}t}$. With $b_{1}\left(t\right)=c_{1}\left(t\right)$ and $b_{2}\left(t\right)=c_{2}\left(t\right)e^{i\Delta t}$, the Schrödinger equation reads

(15) $$i\hbar \left(\begin{array}{c} {\dot{b}}_{1}\left(t\right)\\ {\dot{b}}_{2}\left(t\right) \end{array}\right)={\hat{H}}^{\prime }\left(\begin{array}{c} b_{1}\left(t\right)\\ b_{2}\left(t\right) \end{array}\right),$$

with

(16) $$\begin{array}{c} {\hat{H}}^{\prime }=\left(\begin{array}{cc} 0 & -\frac{\hbar }{2}\Omega_{a}^{*}\\ -\frac{\hbar }{2}\Omega_{a} & -\hbar \Delta \end{array}\right) \end{array}$$

By diagonalizing ${\hat{H}}^{\prime }$, we obtain the eigenvalues $\varepsilon_{\pm }=\frac{\hbar }{2}\left[-\Delta \pm \Omega^{\prime }\right]$ and the corresponding eigenstates

(17) $$|\pm \rangle =\frac{\Omega_{a}^{*}}{\Omega^{\prime }}\sqrt{\frac{\Omega^{{}^{\prime }}}{2[\Omega^{\prime }\mp \Delta ]}}|1\rangle \mp \sqrt{\frac{\Omega^{\prime }\mp \Delta }{2\Omega^{\prime }}}|2\rangle .$$

Under strong pumping of the quantum system–MNP system, the normalized dressed states of the quantum system are given by Equation (17); these are the states interacting with the weak probe field, as schematically depicted in Figure 1b.

## 3. Results and Discussion

### 3.1. Zinc–Phthalocyanine Molecular Complex

**Figure 3:**
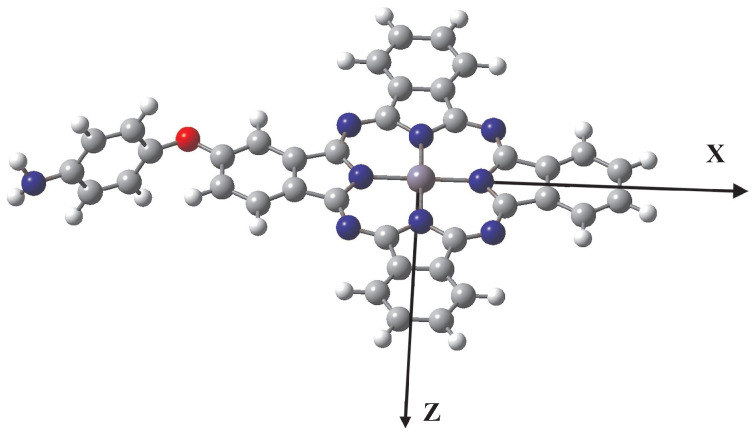
The molecular quantum system used: the zinc–phthalocyanine complex is composed of carbon (gray), hydrogen (white), oxygen (red), nitrogen (blue), and zinc (light blue) atoms. The complex is not planar; however, the phthalocyanine part of the complex is planar, coinciding with the xz-plane, as schematically shown.

In order to compute the linear and nonlinear optical properties of the molecular two-level quantum system in the presence of the MNP, we obtain by ab initio calculations the values of the free-space spectroscopic parameters needed for the zinc–phthalocyanine molecular complex, which is depicted in the graphical representation of Figure 3. The synthesis of this chemical complex has been presented recently [31]. The spectroscopic parameters used in the calculations are obtained by performing geometry optimization of the molecular structure, with respect to state |1〉, the ground electronic state of the molecular complex, at the DFT/B3LYP/6−311$+G^{*}$ level of theory [37], and for state |2〉, the first singlet excited electronic state, by geometry optimization of the molecular structure at the TD-DFT/B3LYP/6-31-$G^{*}$ level [37]. We thus obtain the quantum system transition energy $\hbar \omega_{21}=1.9445$ eV, as we mentioned in the previous section as well as the *x*-oriented transition dipole moment $\mu_{12}=\mu_{21}^{*}=2.9768$ D. We note that the zinc–phthalocyanine molecular complex of this work features permanent dipole moments $\mu_{11}=0.6836$ D and $\mu_{22}=0.7192$ D along the *x*-axis. This makes the molecule weakly polar, since the permanent dipole moments are much smaller than the corresponding transition dipole moment, which allows us to omit their effect on the nonlinear phenomena studied.

### 3.2. Numerical Results

Based on the ab initio obtained spectroscopic parameters, the free-space spontaneous decay rate associated with the |2〉 to |1〉 transition of the organometallic zinc–phthalocyanine molecular complex, $\Gamma^{f}$, is equal to 10.7 $\mu s^{-1}$. In our study, we also assume that the number density of atoms is $N=1.2\times 10^{21}$ m${}^{-3}$. The pump Rabi frequency $|\Omega_{a}^{f}|$ is set equal to 40 μs${}^{-1}$, and the Rabi frequency of the probe field is equal to $|\Omega_{b}^{f}|=0.2$ μs${}^{-1}$. In order to avoid any suppression of the optical effects exhibited by the quantum system, the dephasing rate $\gamma_{d}$ is taken equal to zero.

In the following figures, we present the real and the imaginary part of the susceptibility, as well as the FWM as quantified by ${\left|\rho_{21}^{\left(+\right)}\right|}^{2}$, as a function of the pump–probe frequency mismatch δ, according to Equations (12) and (13), and examine the influence of the distance *d* and the pump field detuning Δ on the optical response of the hybrid system under study.

**Figure 4:**
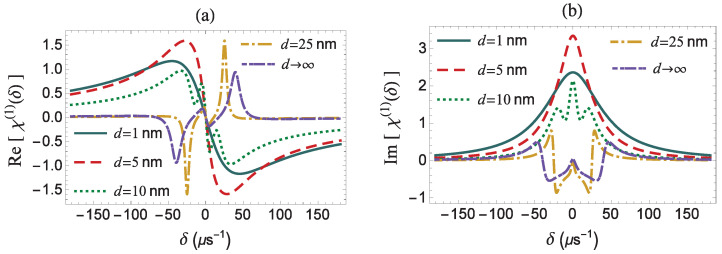
The dispersion (**a**) and absorption (**b**) spectra, as a function of the frequency mismatch of the applied fields δ, for different distances *d* of the quantum system from the surface of the MNP. We assume that $\left|\Omega_{a}^{f}\right|=40\;\mu s^{-1}$, $\gamma_{d}=0$, and Δ=0.

In Figure 4, we present the dispersion and absorption spectra, for various values of the distance between the quantum system and the surface of the MNP, for an exactly resonant pump field (Δ=0). We note that the dispersion spectrum is antisymmetric, while the absorption spectrum is symmetric. The modification of the field modification factor, *f*, as a function of *d* determines the behavior of the effective Rabi frequency and, consequently, the optical response of the quantum system. Two distinct types of dispersion and absorption spectra with quite different characteristics are observed, in the two regions where the conditions $|\Omega_{a}|\;\ll \;\Gamma$ and $|\Omega_{a}|\;\gg \;\Gamma$ hold. The transition between the two different types of the spectra is quite smooth, since the function f(d) does not feature any abrupt change.

**Figure 5:**
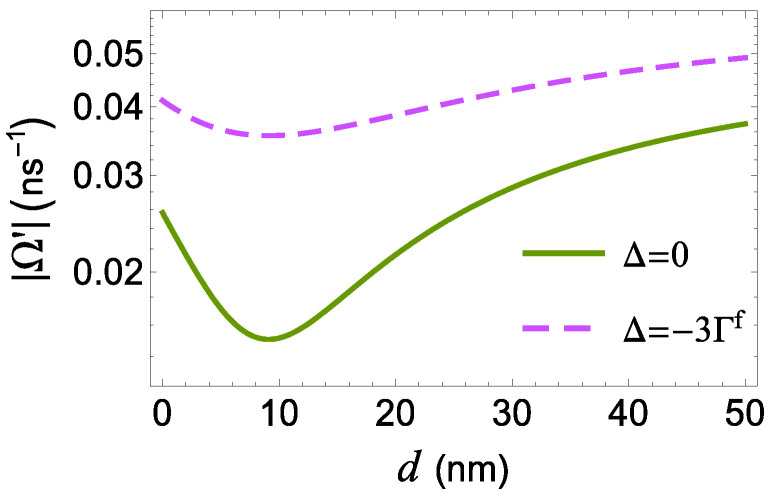
The effective Rabi frequency $|\Omega^{\prime }|$, as a function of the distance *d*, for different values of the detuning Δ. We also take $|\Omega_{a}^{f}|=40\;\mu s^{-1}$ and $\gamma_{d}=0$.

As shown in Figure 5, below the minimum of $\Omega^{\prime }\left(d\right)$ occurring at about *d* = 9.5 nm, which is also the minimum of f(d), both the dispersion and absorption spectra exhibit a single central resonance. Above this critical value, we observe three distinct resonances on their spectral profile. In particular, for d<9.5 nm, we detect a single resonance on the dispersion and absorption spectra, at δ=0, which implies that no efficient energy exchange between the two components of the hybrid structure occurs. The profile of the dispersion and the absorption spectra are typical for a two-level system interacting with a single probe field, since we work in the weak coupling regime with $|\Omega_{a}|\;\ll \;\Gamma$. The profile of the Im$\left[\chi^{\left(1\right)}\left(\delta \right)\right]$ spectrum is Lorentzian-shaped, with a full width at half maximum (FWHM) equal to $\Gamma =g\Gamma^{f}$ and maximal value proportional to the inverse field modification factor, $f^{-1}$. As a result, when the quantum system is located very close to the MNP, as is for d=1 nm (see green solid curve), the FWHM is larger, while the maximal value is smaller as compared to the case with d=5 nm (red dashed curve).

Figure 1b shows a schematic representation of all the possible transition pathways taking place between the dressed states of Equation (17) as well as the corresponding nonlinear processes, for d>9.5 nm. At this point, we proceed to identify the origin of all the three resonances detected on a typical Mollow-type spectrum (see the line shapes of the Re$\left[\chi^{\left(1\right)}\left(\delta \right)\right]$ and Im$\left[\chi^{\left(1\right)}\left(\delta \right)\right]$ spectra presented in Figure 4). The peak at δ=0 is associated with the stimulated Rayleigh scattering process (RL), while the Rabi sidebands at $\delta =\pm |\Omega^{\prime }\left|=\pm f\right|\Omega_{a}^{f}|$ are attributed to the AC-Stark effect (AC) and the three-photon resonance process (TP).

In the region where the *g* factor assumes low values, as it is the case for d=25 nm, in the dispersion spectrum Re$\left[\chi^{\left(1\right)}\left(\delta \right)\right]$, at $\delta =|\Omega_{a}|$, we detect a Lorentzian-shaped sideband resonance, the amplitude of which is inversely proportional to the field modification parameter *f*. The corresponding FWHM equals $\frac{3}{2}g\Gamma^{f}$. When the quantum system is located in free space, (d→∞), both the *g* and *f* factors tend towards 1. As a result, in this case, the FWHM of the sideband resonances and their distance from the spectral center are enhanced. We note that the sideband resonances of the absorption spectrum are dispersion-like, with a slope that is inversely proportional to the field modification factor, *f*. In this region, at d=23 nm, we obtain the maximum gain for the quantum system. In Figure 4b, the yellow dotted-dashed curves correspond to the pump–probe response of the quantum system, when *d* is set equal to 25 nm. If the distance is further increased, see for example the case with d→∞, the gain is suppressed.

**Figure 6:**
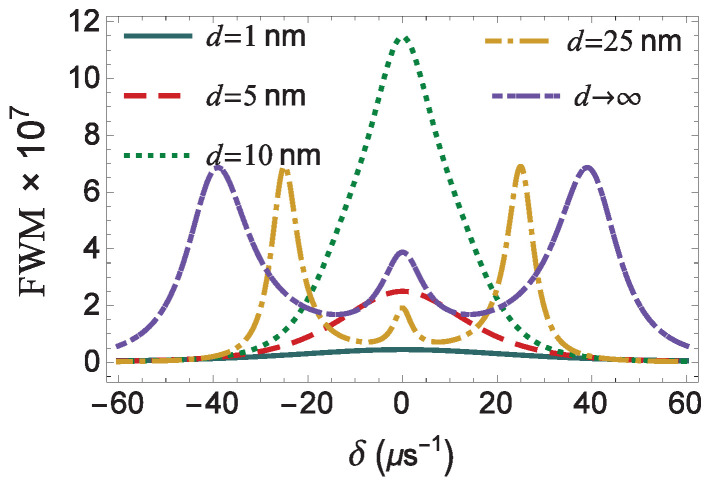
The FWM spectrum, as a function of the frequency mismatch of the applied fields δ, for different distances *d* of the quantum system from the surface of the MNP. We assume that $|\Omega_{a}^{f}|=40\;\mu s^{-1}$, $\gamma_{d}=0$, and Δ=0.

In Figure 6, we present the profiles of the FWM spectra, in the case of a zero pump field detuning (Δ=0), for the same values of the distance as in Figure 4. The profile of the spectrum is symmetric, independent of the value of the distance. In the d<9.5 nm region, where the *g* factor assumes high values, the FWM spectrum exhibits a single resonance that arises at δ=0. Note that the width and the amplitude of this resonance increase monotonically with the spontaneous decay rate modification factor, *g*, and the inverse field modification factor, $f^{-1}$, respectively. In the lower *g*-factor region (d>21 nm), the FWM spectrum is triple-peaked. Here, we observe that the range where the transition from a single-peak to a triple-peak spectral profile takes place is larger compared to the case of the spectra of the pump–probe response (see Figure 4). This implies that, for distance d=10 nm, just 0.5 nm above the characteristic d=9.5 nm value of minimal effective Rabi frequency (Figure 6: green dashed curve), the sideband resonances on the FWM spectrum are not as distinguishable as in the case of the dispersion and the absorption spectra presented in Figure 4. At even larger distances, as is for d=25 nm (yellow dotted-dashed curve), all the three resonance peaks are clearly observable, since now the central resonance is not superimposed with the resonances that emerge at $\delta =\pm |\Omega_{a}|$. For a quantum system placed in free space, i.e., when d→∞, the sideband resonances arise further away from the spectral center compared with the case where we set d=25 nm, because the field modification factor tends to its free-space value (unity), as shown in Figure 2a. These resonances also appear to be broader, due to the amplification of the decay rate modification factor, *g* (see Figure 2b), which, in free-space, becomes equal to one. Here, we also note that, for d=25 nm, the amplitude of the sideband resonances appearing on the FWM spectrum is higher than the corresponding amplitude observed for d→∞. Thus, for intermediate values between the quantum system and the MNP, the optical response of the quantum system is maximized.

**Figure 7:**
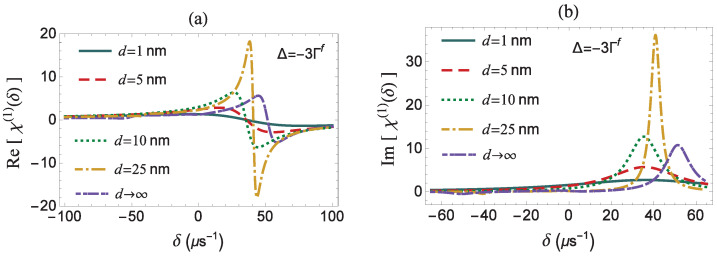
The dispersion (**a**) and absorption (**b**) spectra, as a function of the frequency mismatch of the applied fields δ, for different distances *d* of the quantum system from the surface of the MNP. We assume that $|\Omega_{a}^{f}|=40\;\mu s^{-1}$, $\gamma_{d}=0$, and $\Delta =-3\Gamma^{f}$.

In panels (a) and (b) of Figure 7, we present the dispersion and absorption spectra, respectively, in the case of an off-resonant pump field with a negative pump field detuning $\Delta =-3\Gamma^{f}$, while the rest parameters take the same values as in Figure 4 (exactly resonant pump field). For Δ≠0, the symmetry of the spectra that describe the pump–probe optical response of the coupled quantum system is broken. For negative values of the pump field detuning (Δ<0), the resonance that is detected at the positive side of the δ-axis of the Im$\left[\chi^{\left(1\right)}\left(\delta \right)\right]$ spectrum is dominant, while the one emerging on the negative side is comparatively negligible. More specifically, on the absorption spectrum, we observe a strong absorption Lorentzian-type peak and a weak gain dip, at $\delta =\pm {\left|\Omega \right|}^{\prime }=\pm \sqrt{\Delta^{2}+f^{2}{\left|\Omega_{a}^{f}\right|}^{2}}$, respectively, since the magnitude of the transition dipole moment component associated with the AC Stark effect is larger than the one related to the three-photon resonance transition (see Figure 1), according to Reference [35]. Similarly, a dominant dispersion-like resonance arises on the dispersion spectrum, at $\delta =|\Omega^{\prime }|$. We observe that the prominent resonances (δ>0) are shifted to the right with the increase of *d*. This effect owes its presence to the increase of the field modification factor, *f*, that is responsible for the enhancement of $\left|\Omega^{\prime }\right|$. If we carefully examine the line shapes of the spectra associated with the pump–probe response of the coupled two-level system, while it interacts with an off-resonant pump field, we observe that, for distances lying below a critical value, as in the cases with d=1,5,10 nm, a single resonance is only detected. This behavior is the typical behavior of the two-level system without pumping and is attributed to the fact that, at low interparticle distances, the condition $|\Omega_{a}|\;\ll \;\Gamma$ holds (see Figure 2). Here, the FWHM and the amplitude of the absorption resonance increase monotonically with the *g* and the $f^{-1}$ factors, respectively. We also note that the minimization of the FWHM and the maximization of the amplitude of the predominant spectral resonance on the Im$\left[\chi^{\left(1\right)}\left(\delta \right)\right]$ spectrum both take place in the vicinity of d=23 nm, where the decay rate modification factor, *g*, assumes its minimal value, while, at the same time, the field modification factor, *f*, is not significantly enhanced. In Figure 7, we present the spectra for d=25 nm. In Figure 4, the enhancement of the optical response of the quantum system was also observed for intermediate values of the distance between the two particles. However, for an off-resonant pump field, as in the case studied in Figure 7, we note that this effect is even more pronounced. We also found that, in the case of a positive pump field detuning (Δ>0), the spectra of the pump–probe response are mirror images (with respect to the origin) of the corresponding spectral line shapes obtained for Δ<0, while keeping all the other parameters constant.

**Figure 8:**
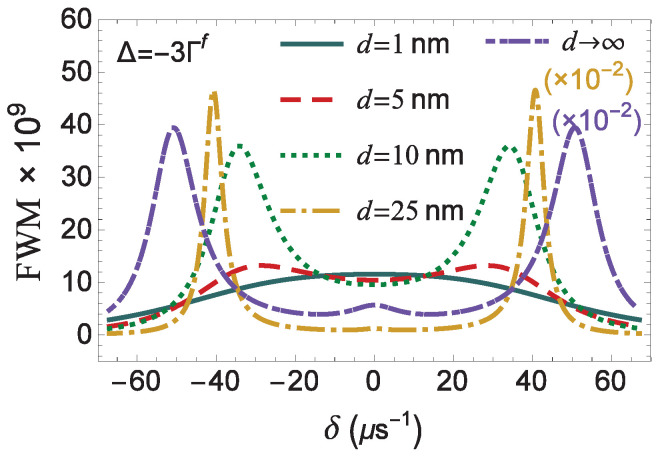
The same as in Figure 6, but with $\Delta =-3\Gamma^{f}$.

In Figure 8, the FWM spectrum is presented for various values of the distance *d*, while the system interacts with an off-resonant pump field with $\Delta =-3\Gamma^{f}$. The profile of the spectrum is symmetric, independent of the value of the distance *d*. For d=1 nm, the FWM spectrum is single-peaked, as in the case of exact resonance (Δ=0) presented in Figure 4b. Practically, for a distance between the quantum system and the surface of the MNP above 5 nm, the FWM spectrum features a doublet of sideband resonances at the positions with $\delta =\pm \sqrt{\Delta^{2}+f^{2}{\left|\Omega_{a}^{f}\right|}^{2}}$. The sideband resonances are shifted away from the center of the FWM spectrum as the distance increases above 9.5 nm, owing its presence to the enhancement of the field modification factor *f*, as seen in Figure 2a. Above 21 nm, a resonance emerges at δ=0 and the spectrum becomes triple-peaked, as in the case with Δ=0. In the region where the spontaneous decay rate modification factor *g* is significantly suppressed, the width of the sideband resonances is minimized, while their amplitude takes a maximal value. This, for example, is observed when we place the quantum system 25 nm away from the surface of the MNP (see Figure 8).

**Figure 9:**
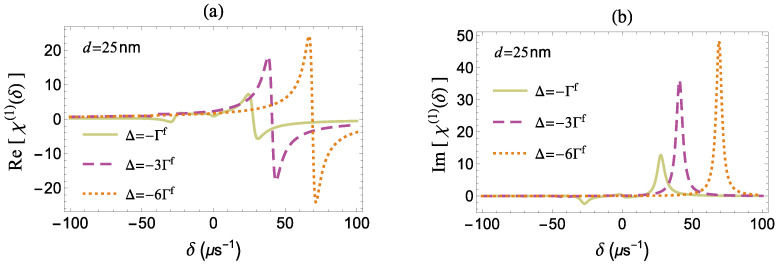
(**a**) The dispersion and (**b**) the absorption spectra versus δ in the presence of the MNP for different Δ with $|\Omega_{a}^{f}|=40\;\mu s^{-1}$, $\gamma_{d}=0$, and d=25 nm.

**Figure 10:**
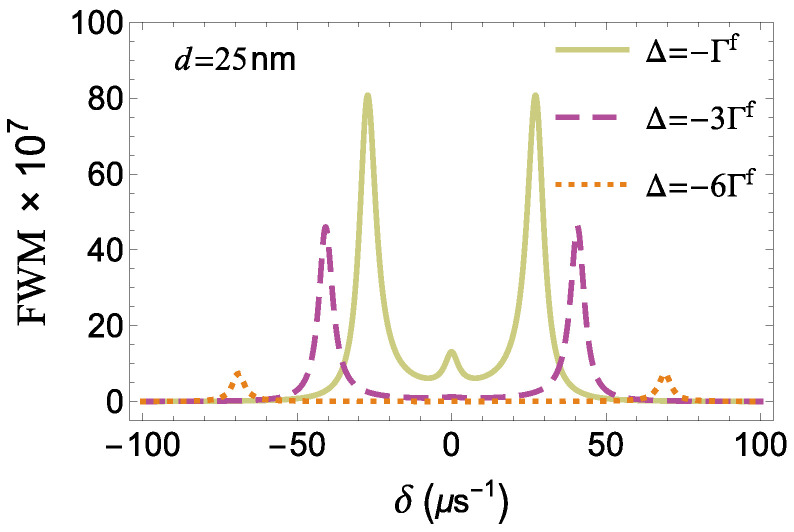
The FWM spectrum as a function of δ in the presence of the MNP for different Δ with $|\Omega_{a}^{f}|=40\;\mu s^{-1}$, $\gamma_{d}=0$, and d=25 nm.

Finally, in Figure 9 and Figure 10, we examine the effect of the pump field detuning Δ on the optical response of the two-level quantum system placed in the vicinity of the MNP, assuming that d=25 nm, where the quantum system exhibits an enhanced optical response, according to the analysis presented in the previous paragraphs. In Figure 9, we note that the asymmetry of the linear dispersion [panel (a)] and absorption [panel (b)] spectra is enhanced as the absolute value of the pump field detuning parameter increases. Moreover, the sideband resonances, which are located at $\delta =\pm |\Omega^{\prime }|=\pm \sqrt{\Delta^{2}+{\left|\Omega_{a}\right|}^{2}}$ based on the dressed state analysis, move away from the center of the spectra with the increase of the |Δ| parameter. This shift may also be explained based on Figure 5. For negative values of Δ, the increase of |Δ| also induces the enhancement (suppression) of the right (left) sideband resonance. In particular, at d=25 nm, we find that the left resonance is practically eliminated for $|\Delta |>6\Gamma^{f}$. As we further enhance the absolute value of Δ, the amplitude of the right resonance increases until a maximal value is obtained that is accompanied by minimal broadening and then exhibits suppression.

As shown in Figure 10, the profile of the FWM spectrum is symmetric for any value of the pump field detuning Δ. As expected, the distance between the sideband resonances $2\sqrt{\Delta^{2}+{\left|\Omega_{a}\right|}^{2}}$ increases with the enhancement of |Δ|. We also observe that, as the absolute value of the pump field detuning increases, the spectrum is gradually suppressed. For $|\Delta |>3\Gamma^{f}$, the central resonance is practically annihilated and, thus, the spectrum becomes double-peaked. For $|\Delta |>6\Gamma^{f}$, the amplitude of the sideband resonances becomes negligible.

To conclude, the value of the field modification factor determines specific characteristics of the FWM spectrum in the following manner. As shown in Figure 2a, *f* is a function of the distance between the quantum system and the surface of the Au-MNP and the Rabi frequency is proportional to the field modification factor. Below the critical distance of 9.5 nm, where the condition $|\Omega_{a}|\;\ll \;\Gamma$ is satisfied, the driving of the molecule is not efficient and the FWM spectrum is single-peaked (see Figure 6 and Figure 8). However, above 9.5 nm, where the condition $|\Omega_{a}|\;\gg \;\Gamma$ holds, there is strong driving of the molecule and the FWM spectrum is triple-peaked. For $|\Omega_{a}|\;\ll \;\Gamma$, we found that the amplitude of the resonance that arises at δ=0 increases monotonically with dependence $f^{-1}$. For $|\Omega_{a}|\;\gg \;\Gamma$, according to the dressed-state analysis, the field modification factor determines the position of the sideband resonances that arise at $\delta =\pm \sqrt{\Delta^{2}+f^{2}{\left|\Omega_{a}^{f}\right|}^{2}}$.

## 4. Conclusions

We studied the pump–probe and the FWM response of a zinc–phthalocyanine molecular quantum system, modeled as a two-level system, in proximity to a spherical gold nanoparticle of relatively large radius. The coupled system interacted with a weak probe field polarized parallel to the surface of the nanoparticle, while the system was pumped with a high-intensity field of the same polarization. The electronic structure used for the molecule in the calculations were obtained by ab initio calculations. The modification of the electric fields and the molecular quantum system spontaneous decay rate, due to the presence of the plasmonic nanoparticle in the vicinity of the quantum system, were obtained by applying an electromagnetic Green’s tensor method. Methodologically, we derived the density matrix differential equations for the dynamics of the two-level quantum system. By a first-order series expansion of the density matrix elements, we obtained analytical expressions for the dispersion, absorption, and FWM spectra of the quantum system. We investigated the dependence of these optical effects on the frequency mismatch between the applied external fields, the detuning of the pump field, and the distance between the quantum system and the surface of the MNP.

We found that the effective Rabi frequency is affected by the field modification factor and the detuning of the strong field. The optical response of the system is also affected by the Purcell factor. The minimum of the effective Rabi frequency function occurs at a distance below which the quantum system spectra are single-peaked, as in the case of a two-level system interacting with a single probe field. The width and the amplitude of this resonance increases monotonically with the spontaneous decay rate modification factor and the inverse field modification factor, respectively. Above the critical value, the increase of the distance between the quantum system and the MNP induces a shift of the sideband resonances away from the spectral center, due to the enhancement of the field modification factor, as well as their broadening, due to the amplification of the decay rate modification factor. The absorption/dispersion spectra exhibit the typical Mollow-type profile, since the field enhancement factor increases, while the spontaneous decay rate modification factor decreases and the FWM spectrum becomes triple-peaked.

In the case of an off-resonant pump field, the symmetry of the dispersion and the absorption spectrum is broken, while the FWM spectrum remains symmetric, as in the case of exact resonance. As the absolute value of the pump field detuning increases, the distance between the sideband resonances is enhanced. At the same time, the asymmetry of the dispersion and absorption spectra is intensified, and the predominant resonances reach a maximum value before experiencing suppression, while the FWM spectrum decreases monotonically. Finally, and most importantly, we discovered that the optical response of the coupled quantum system is maximized for an intermediate value of the interparticle distance, where the decay rate modification factor presents an important suppression; the enhancement of the absolute value of the pump field detuning further reinforces the optical response of the quantum system.

Before closing the article, we would like to mention that a different MNP in shape or a core–shell type of nanoparticle may give different results, as the results depend on the modification factor *f* and the Purcell factor *g*, which, for these nanoparticles will be different. The material of the nanoparticle also has an important effect. We do not claim that the spherical Au nanoparticle is the optimal MNP compared to other types. It is simply the most commonly studied MNP which is routinely fabricated in the lab for many years. For this reason, we have decided to start our studies with this one. The effect of other MNPs on the presented results is a matter of investigation that is beyond the scope of the current paper, but it is certainly of interest.

## Data Availability

The data presented in this study are available upon reasonable request from the corresponding author.

## Abbreviations

The following abbreviations are used in this manuscript:

B3LYP	Becke, 3-parameter, Lee–Yang–Parr
DFT	Density Functional Theory
FWM	Four-Wave Mixing
MNP	Metallic Nanoparticle
TD-DFT	Time-Dependent Density Functional Theory
